# Structural Deficit-Guided Reconstruction of an Extended Lower Eyelid–Bicanthal Defect

**DOI:** 10.3390/jcm15176844

**Published:** 2026-09-03

**Authors:** Zheng-Cai Wang, Zi-Chun Gu, Ying Hu, Jia-Qin Cai, Hua Li

**Affiliations:** Department of Plastic Surgery, Sir Run Run Shaw Hospital, Zhejiang University School of Medicine, Hangzhou 310016, China; wangzhengcai@zju.edu.cn (Z.-C.W.);

**Keywords:** lower eyelid reconstruction, bicanthal defect, individualized reconstruction, septal chondromucosal graft, midface lift, bipedicled upper eyelid flap

## Abstract

**Background/Objectives:** Extended lower eyelid defects involving both lamellae and canthal supports require restoration of lining, support, vascularized coverage, and horizontal stability. We describe a two-stage reconstruction guided by structural deficits and available autologous tissues. **Methods:** A 63-year-old man underwent staged excision with frozen-section margin control for conventional-type basal cell carcinoma involving almost the entire right lower eyelid. A lengthened septal chondromucosal graft restored posterior support and lining. A right transtemporal midface lift and a near-margin bipedicled upper eyelid orbicularis myocutaneous flap provided anterior coverage. Six months later, the lateral pedicle was divided, the graft was contoured and fixed transosseously to the lateral orbital rim, and the medial pedicle tunnel was revised. **Results:** All transferred tissues survived. At final follow-up, MRD2 measured 4.61 mm on the reconstructed side and 4.12 mm contralaterally (difference, 0.49 mm); palpebral fissure height measured 6.02 and 5.92 mm, respectively (difference, 0.10 mm). Lower scleral show and the residual gap during forceful closure were 0 mm. The absolute lateral canthal height difference was 1.45 mm preoperatively, 4.14 mm after stage 1, 0.45 mm after stage 2, and 1.58 mm at final follow-up. At 4 years, globe apposition was maintained without ectropion, entropion, retraction, or graft exposure; magnetic resonance imaging showed no recurrence. **Conclusions:** This case illustrates a potentially transferable process: define structural and functional deficits first, then match each reconstructive objective to suitable patient-specific tissues.

## 1. Introduction

The lower eyelid is a composite structure. The posterior lamella, formed by the tarsus and palpebral conjunctiva, provides support and a smooth globe-facing surface. The anterior lamella, formed by skin and orbicularis oculi, provides vascularized coverage and contributes to eyelid closure. The medial and lateral canthal structures maintain horizontal stability and lid–globe apposition. Simultaneous loss of these components cannot be corrected by coverage alone [1,2,3].

Successful reconstruction requires coordinated restoration of posterior support and lining, vascularized anterior coverage, and canthal anchoring. Because several techniques can satisfy each requirement, selection should be individualized according to the defect and the tissues available [1,2,4,5,6].

No single method is optimal for every patient. Technique selection should reflect the structures and functions lost, the patient’s available donor tissues, donor-site morbidity, and the aesthetic consequences of each approach. We applied this principle to an extended lower eyelid–bicanthal defect with complete loss of the lower tarsus, palpebral conjunctiva, eyelid margin, and both canthal supports. The aim was to describe the decision process, operative sequence, objective photographic outcomes, and 4-year clinical result of this individualized reconstruction.

## 2. Materials and Methods

### 2.1. Study Design, Ethics, and Follow-Up

This retrospective single-patient technical study was conducted in accordance with the Declaration of Helsinki and was approved by the Institutional Review Board of Sir Run Run Shaw Hospital, Zhejiang University School of Medicine (approval number: 2026-2672-01; approval date: 17 July 2026). The patient provided written informed consent for treatment and for publication of identifiable clinical photographs, magnetic resonance images, intraoperative photographs, and video materials.

Clinical records, operative notes, frozen-section and permanent histopathology reports, serial photographs, ocular motility examination records, and magnetic resonance imaging were reviewed. Outcomes were evaluated preoperatively, 6 months after first-stage reconstruction (immediately before the second stage), 6 months after second-stage reconstruction, and at final follow-up 4 years after the first stage and 3.5 years after the second stage.

### 2.2. Oncologic Resection and Defect Assessment

**Figure 1:**
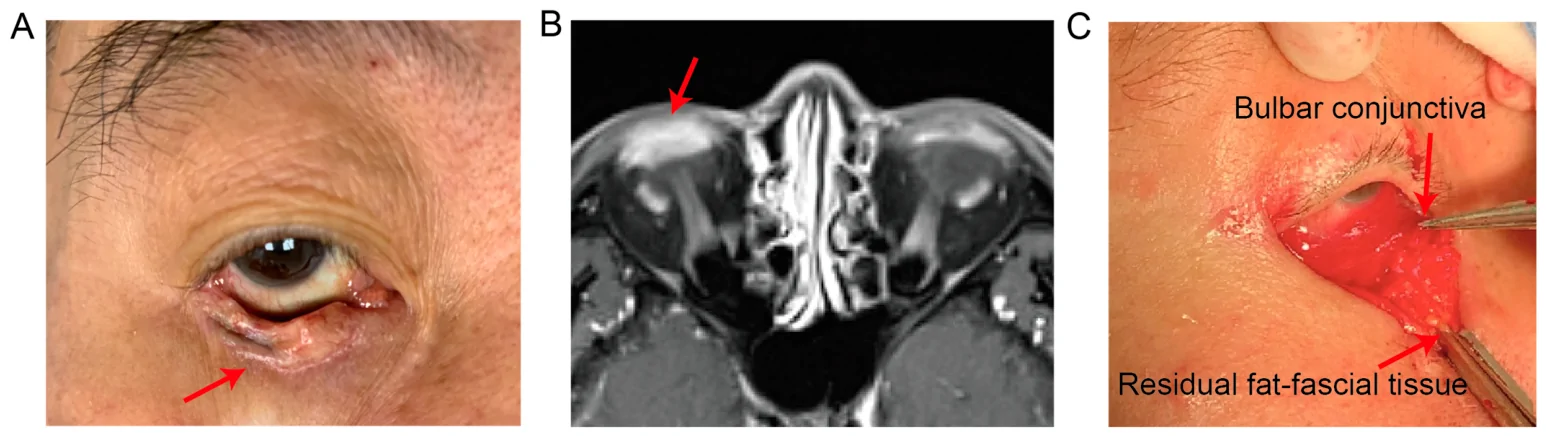
Tumor extent and postexcisional defect. (**A**) Clinical appearance of the lower eyelid tumor (red arrow). (**B**) Enlarged preoperative MRI view of the tumor region (red arrow). (**C**) Full-thickness lower eyelid–bicanthal defect; the medial forceps indicates the residual bulbar conjunctiva, whereas the lateral forceps indicates residual orbital fat and fascial tissue at the resection margin.

A 63-year-old man presented with basal cell carcinoma involving almost the entire right lower eyelid. Clinical examination defined the visible tumor extent (Figure 1A). Preoperative magnetic resonance imaging demonstrated the local extent while supporting preservation of the globe and extraocular muscles (Figure 1B).

The tumor was removed by staged excision with intraoperative frozen-section margin control. Three rounds of margin assessment were required. Final frozen sections were negative. Permanent paraffin-embedded histopathology confirmed conventional-type basal cell carcinoma with negative margins.

After excision, the defect encompassed the entire lower eyelid margin and lashes, the complete lower tarsus, the palpebral conjunctiva from the former lower eyelid margin to the inferior fornix, and the anterior lamella, including part of the orbicularis oculi. Both canthal supports, the caruncle, lower lacrimal punctum, lower canaliculus, and lacrimal sac were also resected, leaving no reliable native canthal anchor. The bulbar conjunctiva, cornea, globe, and extraocular muscles were preserved (Figure 1C and Video S1).

### 2.3. Patient-Specific Reconstructive Planning

Planning began with the structural deficits rather than a predetermined flap. For each missing component, the required function was defined and then matched to a patient-specific tissue resource. The first stage was designed to restore posterior lining and support, recruit vascularized anterior tissue, and obtain stable coverage. The second stage was reserved for definitive lateral fixation and contour refinement after tissue integration. The decision matrix is summarized in Table 1.

### 2.4. First-Stage Reconstruction

#### 2.4.1. Posterior Lamellar Reconstruction

Complete loss of the lower tarsus and palpebral conjunctiva required structural support and a smooth globe-facing lining. A tarsoconjunctival flap would have used upper eyelid posterior lamella and temporarily occluded the visual axis [4,5,6], whereas cartilage alone would have required a separate lining [1,7].

**Figure 2:**
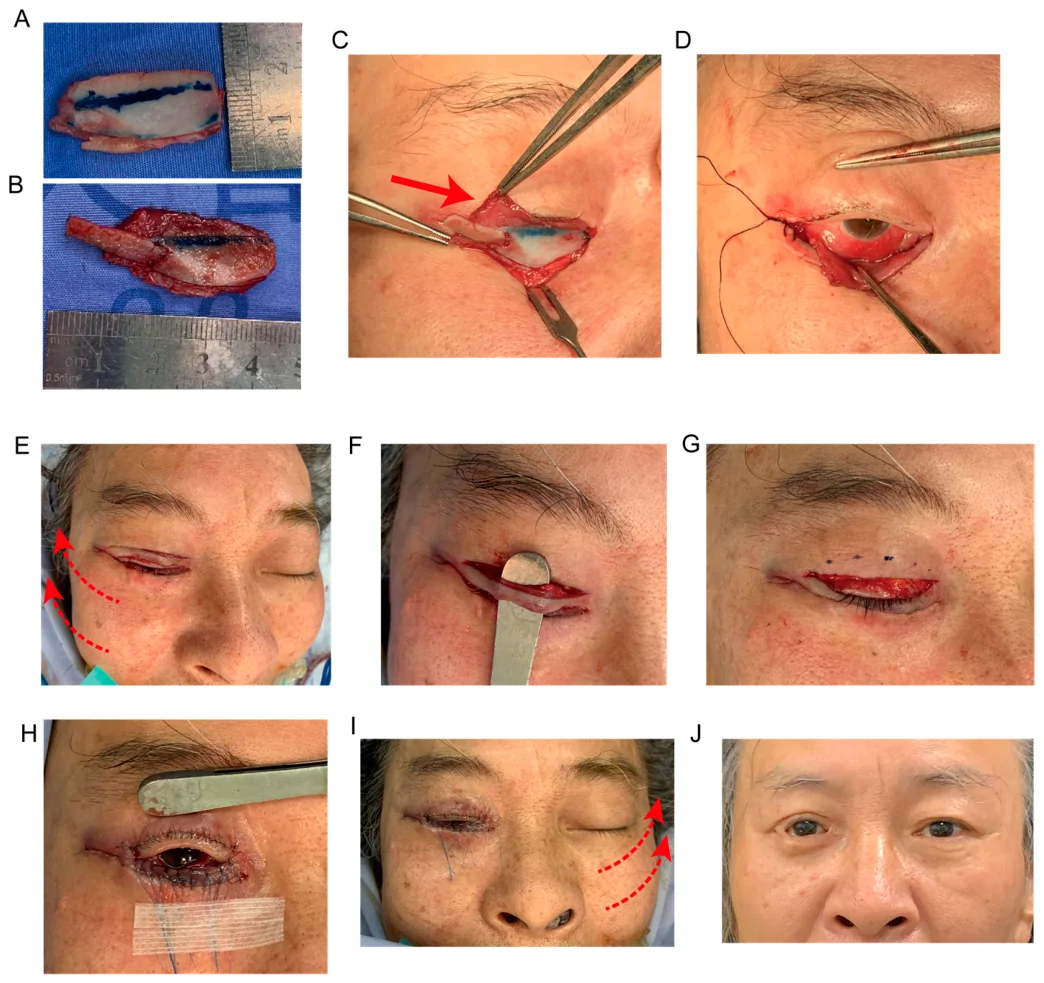
First-stage reconstruction. (**A**) Septal chondromucosal graft, 3.5 × 1.2 cm. (**B**) Lengthened graft, 4.5 × 1.2 cm, with preserved mucosal continuity. (**C**,**D**) Graft placement; the red arrow in (**C**) indicates the graft. (**E**) Right transtemporal midface lift; the red dashed arrow indicates superolateral advancement. (**F**) Near-margin bipedicled upper eyelid orbicularis myocutaneous flap. (**G**) Inferior flap transfer. (**H**) Immediate right lower eyelid result. (**I**) Appearance after left midface lift. (**J**) Six-month result showing pedicle tethering, lateral graft prominence, and a medial pedicle tunnel.

The patient had an adequately developed nasal septum. A septal chondromucosal graft was therefore selected because it supplied cartilage and mucosa in one unit, while the donor scar remained intranasal [7,8,9]. The harvested graft measured 3.5 × 1.2 cm (Figure 2A). Its length was insufficient for the 4.5 cm defect. A cartilage segment was partially divided and repositioned, while mucosal and perichondrial continuity was preserved. The cartilage segments were secured with polydioxanone sutures, producing a 4.5 × 1.2 cm graft (Figure 2B).

No thinning or scoring was performed during the first stage. The mucosal surface was oriented toward the globe and sutured to the residual conjunctiva. The graft formed the reconstructed posterior lamella and new lower eyelid margin (Figure 2C,D). Because both native canthal tendons were absent, the medial and lateral graft ends were provisionally secured to the adjacent residual soft tissues.

#### 2.4.2. Anterior Lamellar Reconstruction with a Transtemporal Midface Lift

Loss of lower eyelid skin and part of the orbicularis produced a broad anterior defect. A free skin graft would have placed one nonvascularized graft over another without an interposed vascularized layer. It would also have provided no orbicularis tissue or vertical recruitment. A larger cheek rotation flap was possible but would have required wider facial dissection and a less concealed scar pattern.

The patient had marked age-related laxity of the midface soft tissues. A right temporal incision was placed within the hair-bearing scalp. The midface skin–orbicularis unit was elevated in the superficial temporal fascia/superficial musculoaponeurotic system plane, advanced superolaterally, and suspended to the deep temporal fascia (Figure 2E). This maneuver covered most of the chondromucosal graft, reduced the vertical defect, and moved viable orbital and preseptal orbicularis toward the reconstructed lower eyelid. The concealed incision limited visible scarring and repositioning of the lax tissues improved the eyelid–cheek contour.

After the midface lift, a residual anterior lamellar gap of approximately 3 mm remained adjacent to the new lower eyelid margin. A separate thin, vascularized tissue layer was therefore required.

#### 2.4.3. Near-Margin Bipedicled Upper Eyelid Flap

The patient had sufficient redundant upper eyelid skin to permit harvest of a 5 mm-wide skin–orbicularis flap, allowing tension-free coverage of the approximately 3 mm residual defect. Conventional Tripier-type flaps are generally designed at or near the supratarsal crease [1]. In this patient, that level would have required a longer inferior transfer and greater rotation of both pedicles; a near-margin design shortened the transfer and reduced pedicle torsion.

A bipedicled upper eyelid orbicularis myocutaneous flap measuring 4.5 cm in length and up to 5 mm in width was designed immediately above the upper eyelid margin (Figure 2F). It contained skin and pretarsal orbicularis. Dissection remained anterior to the tarsus to protect the tarsus and posterior tarsal vascular supply. The upper eyelid margin, eyelashes, tarsus, and orbital septum were preserved. Medial and lateral pedicles of approximately 4 mm were retained.

The pedicles maintained flap perfusion and created temporary tissue bridges across the absent canthal regions. The flap was turned inferiorly to cover the remaining anterior defect and exposed graft (Figure 2G). Its superior edge after transfer was sutured to the anterior graft margin, where a narrow mucosal cuff had been preserved. The flap orbicularis was coapted to the advanced orbital and preseptal orbicularis.

The flap design incorporated part of the redundant upper eyelid skin and enabled formation of the right upper eyelid crease through the palpebral-margin incision using the technique previously reported by our group [10]. The immediate result is shown in Figure 2H. A left transtemporal midface lift was subsequently performed for facial balance (Figure 2I); it did not contribute to the blood supply of the reconstructed right lower eyelid.

**Figure 3:**
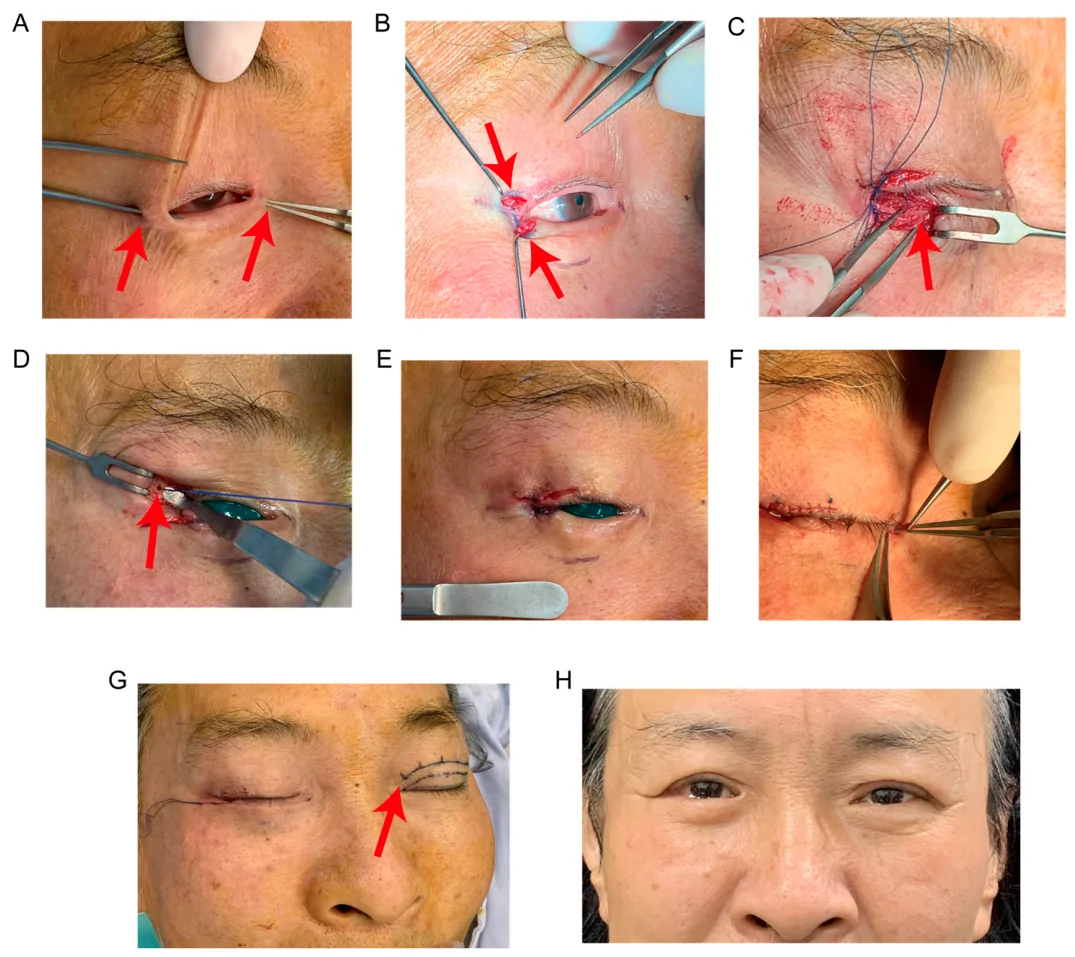
Second-stage refinement. (**A**) Pedicles before division; the red arrow indicates the medial cutaneous tunnel, and the lateral pedicle produces inferior traction. (**B**) Divided lateral pedicle ends (red arrow). (**C**) Redundant lateral graft tissue (red arrow). (**D**) Transosseous fixation at the lateral canthal level (red arrow). (**E**) Lateral canthoplasty. (**F**) Medial tunnel revision. (**G**) Immediate result; the red arrow indicates the left palpebral-margin crease design. (**H**) Six-month result.

At 6 months, all transferred tissues had survived. The temporary tissue bridges limited early displacement but produced angular tethering at the medial and lateral pedicles. The lateral part of the graft remained prominent, and an epithelialized cutaneous tunnel persisted around the medial pedicle (Figure 2J and Figure 3A). These findings defined the objectives of the second stage.

### 2.5. Second-Stage Refinement and Definitive Lateral Fixation

The lateral pedicle was divided after tissue integration to release lateral tethering and permit lateral canthal reconstruction (Figure 3B). The prominent lateral part of the graft was then exposed, thinned, and contoured to improve globe conformity (Figure 3C).

After pedicle division, the reconstructed eyelid required definitive lateral support. The intended lateral canthal level was determined from the contralateral side. A bony tunnel was drilled through the lateral orbital rim at the canthal level. A nonabsorbable silk suture was selected to provide a permanent, firm, and reliable fixation point; it was passed through the tunnel and the lateral end of the cartilage graft and buried beneath the soft tissue. The graft was secured under controlled horizontal tension (Figure 3D). The reconstructed lateral canthus was positioned slightly higher than the medial canthus, and lateral canthoplasty was completed (Figure 3E). Transosseous or periosteal fixation provides a stable vector when native lateral support is absent [11,12].

The medial side was treated differently. The medial canthal tendon and lacrimal sac had been completely resected, leaving no usable tendon stump. Recent reconstructive options include autogenous fascia lata grafts, hinged tarsal flaps, and micro-anchor fixation [13,14,15]. In this patient, these alternatives would have required additional donor tissue, use of upper eyelid tarsal tissue, or deeper dissection and implanted fixation in an already extensively resected medial canthal region. Because the medial pedicle remained viable and maintained adequate soft-tissue continuity, additional medial bone fixation was not selected. The epithelialized tunnel was opened, de-epithelialized, trimmed, and fixed to the underlying bed while the deep pedicle was preserved (Figure 3F).

The right upper eyelid crease had stabilized. A left double-eyelid procedure through a palpebral-margin incision was performed during the same operation to improve bilateral symmetry (Figure 3G) [10]. The 6-month result is shown in Figure 3H.

### 2.6. Photographic Measurements and Ophthalmic Assessment

Serial frontal photographs obtained in primary gaze and during maximal forceful eyelid closure were retrospectively analyzed using Fiji (ImageJ 1.52a; National Institutes of Health, Bethesda, MD, USA). Images were calibrated using the bilateral horizontal white-to-white corneal diameter (HWTW) of 11.3 mm as the internal scale. HWTW was automatically measured from anterior-segment iris images acquired with the OCULUS Pentacam system. Images with obscured landmarks, marked head rotation or tilt, or nonprimary gaze were excluded from the corresponding measurement; all selected images permitted identification of the required landmarks.

Measurements included MRD2, lower scleral show, palpebral fissure height, lateral canthal height, the absolute bilateral lateral canthal height difference, and the maximum residual vertical gap during forceful closure. Two researchers performed the measurements independently. Each researcher measured every indicator three times on each photograph, and the three readings were averaged for that researcher; the two researcher means were then averaged to obtain the reported value. Intraobserver and interobserver reliability were not formally assessed. 

Formal ocular motility examinations were performed at each postoperative follow-up. Dry-eye symptoms, recurrent conjunctivitis, and the need for long-term ocular lubrication were assessed from the clinical records and patient reports. No standardized slit-lamp ocular-surface examination, Schirmer test, tear break-up time measurement, or corneal staining was performed. At final follow-up, the patient completed the official Simplified Chinese (China) version of four independently functioning scales from the validated FACE-Q Skin Cancer Module: Satisfaction with Facial Appearance, Appraisal of Scars, Appearance-Related Distress, and Satisfaction with Information: Appearance [16]. Raw summed scores were converted to 0–100 Rasch-transformed scores using the corresponding conversion tables. Higher scores indicate greater satisfaction or a more favorable scar appraisal for the three satisfaction/appraisal scales, whereas higher Appearance-Related Distress scores indicate greater distress. No preoperative patient-reported outcome measure was available. Because this was a single-patient study, the scores were reported descriptively without longitudinal comparison or inferential statistical analysis.

## 3. Results

### 3.1. Tissue Survival and Early Complications

The septal chondromucosal graft, tissues mobilized during the bilateral transtemporal midface lifts, and the bipedicled upper eyelid flap survived. There was no necrosis, infection, wound dehiscence, or graft exposure. The right upper eyelid donor site healed without eyelid-margin deformity, retraction, closure disturbance, or lash loss. At the nasal septal donor site, there was no septal perforation, significant bleeding, infection, persistent airway or nasal-obstruction symptom, or external nasal contour change. Final-follow-up nasal endoscopy showed a healed septal mucosal incision without obvious internal deformity or visible hypertrophic scarring (Figure S1).

At 6 months after stage 1, the main limitations were pedicle-related angular tethering, inferior traction at the lateral pedicle, lateral graft prominence, and a medial pedicle cutaneous tunnel. These findings were corrected or reduced during the planned second stage.

### 3.2. Serial Photographic Measurements

Serial measurements are shown in Table 2. Preoperative values on the affected side reflected tumor-related distortion. At 6 months after stage 1, right/left MRD2 values were 4.78/4.20 mm (difference, 0.58 mm); at 6 months after stage 2, they were 4.20/3.94 mm (difference, 0.26 mm); and at final follow-up, they were 4.61/4.12 mm (difference, 0.49 mm). Right lower scleral show measured 1.66 mm preoperatively and 0 mm at each postoperative assessment. The absolute lateral canthal height difference measured 1.45 mm preoperatively, 4.14 mm at 6 months after stage 1, 0.45 mm at 6 months after stage 2, and 1.58 mm at final follow-up. Final palpebral fissure height measured 6.02 mm on the reconstructed side and 5.92 mm contralaterally (difference, 0.10 mm). The residual vertical gap during forceful closure was 0 mm at all four assessments.

MRD2 was measured vertically from the corneal light reflex to the lower eyelid margin. Lower scleral show was measured from the inferior corneal limbus to the lower eyelid margin. Palpebral fissure height was measured through the pupil center. Lateral canthal height was measured relative to the line connecting the medial canthi; positive values indicate a position above the line and negative values indicate a position below it. Bilateral lateral canthal height difference is the absolute difference between sides. The maximum residual vertical gap was measured during forceful eyelid closure. Reported values are the average of the two researcher means; each researcher mean was based on three repeated measurements. All values were calibrated using the bilateral HWTW of 11.3 mm, which was automatically measured from anterior-segment iris images acquired with the OCULUS Pentacam system. Preoperative affected-side measurements reflected tumor-related distortion and were not normal baseline anatomy.

### 3.3. Long-Term Functional and Oncologic Outcomes

**Figure 4:**
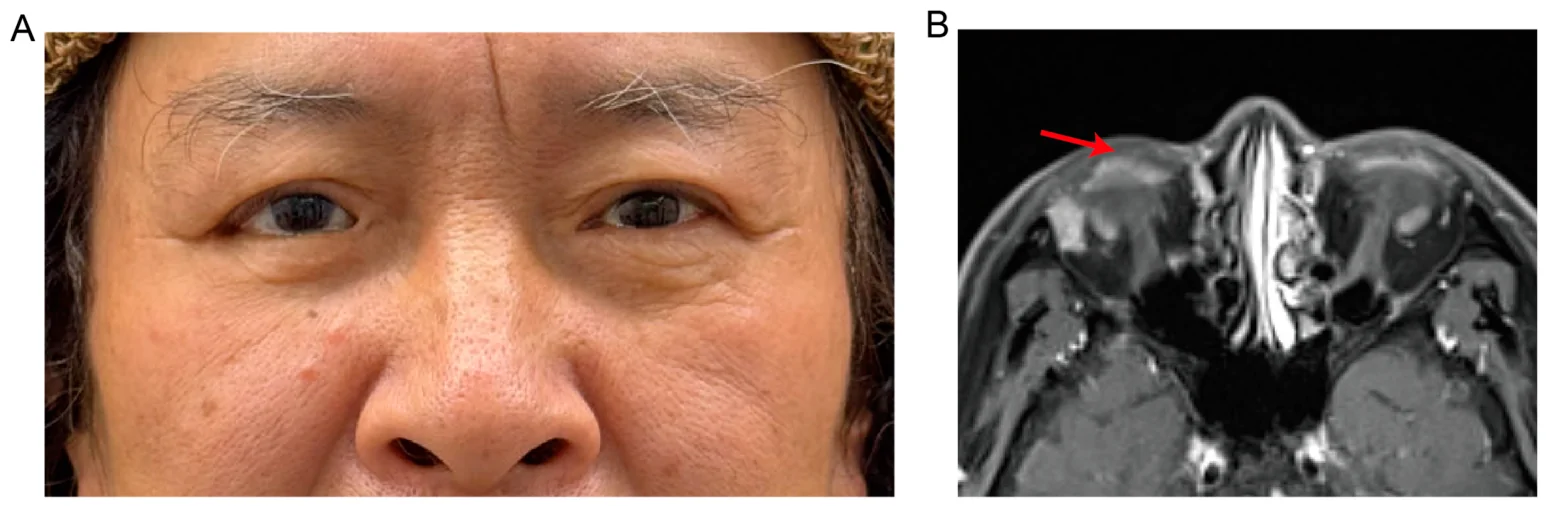
Long-term outcome. (**A**) Four years after stage 1 and 3.5 years after stage 2. (**B**) Enlarged follow-up MRI view of the prior tumor bed/reconstructed lower-eyelid region; the red arrow indicates the retained cartilage graft, and no recurrence was identified.

At final follow-up, the reconstructed lower eyelid maintained globe apposition and complete eyelid closure (Figure 4A). There was no ectropion, entropion, lower eyelid retraction, recurrent graft prominence, or graft exposure. Formal ocular motility examination showed no diplopia, ocular misalignment, or clinically apparent extraocular movement restriction. The patient reported no dry-eye symptoms, had no documented recurrent conjunctivitis, and did not require long-term artificial tears or ocular lubricants. No late silk-suture-related granuloma, infection, extrusion, palpability, or other suture-related complication was observed. In the final-follow-up-only FACE-Q assessment, Satisfaction with Facial Appearance, Appraisal of Scars, and Satisfaction with Information: Appearance were each 100/100, and Appearance-Related Distress was 0/100. These cross-sectional scores indicate the most favorable response pattern at that assessment; they cannot be interpreted as preoperative-to-postoperative change. Satisfaction with Information: Appearance reflects the patient’s experience of appearance-related information rather than the reconstructive result itself.

Persistent epiphora remained after resection of the lower punctum, lower canaliculus, and lacrimal sac. Lacrimal reconstruction was considered during the initial planning, but conventional canalicular repair and dacryocystorhinostomy could not restore the completely absent lower drainage pathway. Oncologic clearance, globe protection, vascularized coverage, and stable bicanthal reconstruction were prioritized, while a bypass procedure was reserved as a secondary option. At follow-up, the patient considered the epiphora acceptable, was satisfied with the reconstruction, and declined further lacrimal surgery. Magnetic resonance imaging demonstrated the retained cartilage graft and no tumor recurrence (Figure 4B).

## 4. Discussion

The principal contribution of this case is not a new individual graft or flap. It is the sequence used to match each structural deficit to an available patient-specific tissue resource. The posterior defect required support and ocular lining. The anterior defect required a vascularized layer, vertical tissue recruitment, and partial restoration of orbicularis continuity. Loss of both canthal supports required early positional control followed by definitive horizontal fixation.

The septal graft was selected because adequate cartilage and mucosa could be obtained through a concealed intranasal donor site [7,8,9]. Cartilage rearrangement compensated for the limited harvest length while maintaining a continuous globe-facing mucosal layer. The choice and modification were therefore dictated by this defect and the patient’s donor anatomy.

The anterior plan used the patient’s midface soft-tissue laxity and upper eyelid skin redundancy. The midface lift recruited vascularized skin and orbicularis through a concealed scalp incision. Although the residual marginal gap measured approximately 3 mm, a flap up to 5 mm wide was selected to permit tension-free inset and reduce downward traction that could promote lower eyelid eversion. The upper eyelid had sufficient redundant skin to tolerate this width without donor-site shortage, and contralateral lower eyelid skin redundancy made the modest reconstructed fullness relatively symmetric. The near-margin position also shortened transfer and reduced pedicle rotation compared with a supratarsal design.

Loss of both canthal supports required staged positional control. The flap pedicles acted as temporary vascularized bridges rather than reconstructed tendons. Their traction increased the absolute lateral canthal height difference from 1.45 mm preoperatively to 4.14 mm at 6 months after stage 1. After tissue integration, lateral pedicle division and transosseous fixation reduced the difference to 0.45 mm at 6 months after stage 2; the difference was 1.58 mm at final follow-up. Single-stage reconstruction is feasible for many large lower eyelid defects: Kwon et al. [17] reported 40 patients treated with a customized midface lift, with or without a free mucochondral graft. In the present case, however, the defect also included complete loss of both canthal supports without a usable tendon stump. The first stage provided vascularized coverage and temporary positional control while the graft and flap integrated; the second stage allowed pedicle division, graft contouring, and definitive lateral transosseous fixation. This choice reflects the additional canthal reconstruction requirement and does not imply superiority over single-stage approaches.

This combination is most applicable to a near-total full-thickness lower eyelid defect with simultaneous loss of posterior lamellar lining and support, anterior vascularized coverage, the eyelid margin, and both canthal supports, particularly when no reliable tendon stump remains. Patient factors favoring the strategy include adequate septal chondromucosa, midface soft-tissue laxity, sufficient upper eyelid skin redundancy, preserved residual conjunctiva and orbicularis, an intact lateral orbital rim for delayed fixation, and willingness and fitness for staged surgery. Smaller defects, preserved canthal stumps, adequate local tarsoconjunctiva, limited donor tissue, or a need for single-stage reconstruction may favor more conventional alternatives.

The lateral and medial sides were not treated identically. The intact lateral orbital rim provided a strong, accessible site for transosseous fixation [11,12]. Medially, no tendon stump remained after resection of the medial canthal tendon and lacrimal sac. The alternative options noted above would have added donor-site morbidity, use of upper eyelid tarsal tissue, or implanted fixation and deeper dissection near the resected lacrimal fossa [13,14,15]. Because the surviving medial pedicle maintained acceptable continuity and position, it was preserved and only the epithelialized tunnel was revised. This was a patient-specific compromise rather than an anatomic recreation of the medial canthal tendon.

Lacrimal reconstruction options were considered during the initial planning. Conventional canalicular repair was not feasible because the lower punctum and lower canaliculus were completely absent, and conventional dacryocystorhinostomy could not use the resected lacrimal sac. Canaliculorhinostomy can create drainage in selected patients without a lacrimal sac, but reported techniques require an adequate intact canalicular remnant, a bony ostium, nasal mucosal work, and temporary intubation; tube prolapse, ostial synechia, granulation, nasal bleeding, dryness, and olfactory dysfunction have been reported [18,19]. Conjunctivodacryocystorhinostomy with a Jones tube can bypass deficient canaliculi, but it introduces a permanently maintained transnasal prosthesis with risks of extrusion, malposition, infection, obstruction, granuloma, and medial canthal deformity [20]. In this patient, adding medial canthal and endonasal dissection or a permanent tube during extensive bicanthal reconstruction could have increased morbidity and disturbed the reconstructed medial contour and support. Lacrimal bypass was therefore reserved as a secondary option; the patient later found the epiphora acceptable and declined further surgery.

Aesthetic considerations were incorporated only after oncologic clearance and the essential functional requirements had been secured. The intranasal donor site, temporal scalp incisions, and palpebral-margin incisions concealed most scars. Midface repositioning and upper eyelid crease formation also improved facial balance. These benefits were secondary to globe protection, eyelid closure, and stable lid–globe apposition.

This study has several limitations. It describes one patient and cannot establish comparative superiority. Photographic measurements are sensitive to camera position, gaze, head rotation, calibration, and landmark identification; small perspective differences may remain despite patient-specific HWTW calibration. Although two researchers performed triplicate measurements, intraobserver and interobserver reliability were not formally assessed. The contribution of orbicularis coaptation was not assessed with electromyography or dynamic imaging. Ocular-surface outcomes were based on symptoms and clinical records; standardized slit-lamp ocular-surface examination, Schirmer testing, tear break-up time, and corneal staining were not performed. The four FACE-Q Skin Cancer Module scales were added only at final follow-up; the cross-sectional scores therefore cannot quantify change or fully represent functional reconstructive outcomes. Satisfaction with Information: Appearance evaluates the patient experience of counseling rather than the surgical result, and the most favorable response pattern may be affected by a ceiling effect. The two-stage plan required temporary tethering, graft prominence, and a second operation. Persistent epiphora remained because the lower lacrimal drainage pathway was not reconstructed.

## 5. Conclusions

Extended lower eyelid reconstruction should begin with the missing structures and their functional consequences. Available methods should then be matched to the patient’s anatomy, donor tissues, anticipated morbidity, and aesthetic needs.

The underlying decision principle may be applicable to other complex lower eyelid defects, whereas the operative combination should remain patient-specific. In this patient, a septal chondromucosal graft restored posterior support and lining, a transtemporal midface lift recruited vascularized anterior tissue, a near-margin bipedicled upper eyelid flap completed coverage, and delayed transosseous fixation restored lateral stability.

## Figures and Tables

**Table 1:** Deficit-based reconstructive planning.

Structural Deficit	Functional Need	Available Patient Factor	Selected Method
Posterior lamella	Support and lining	Adequate septum	Lengthened septal graft
Anterior lamella	Vascularized coverage	Midface laxity	Transtemporal midface lift
Marginal gap	Thin, tension-free coverage	Upper-lid skin redundancy	Bipedicled upper-lid flap
Canthal supports	Horizontal stability	No tendon stumps; intact lateral rim	Temporary bridges; delayed lateral fixation

**Table 2:** Serial eyelid measurements.

Outcome	Preoperative	6 Months After Stage 1	6 Months After Stage 2	Final Follow-Up
MRD2, right/left (mm)	7.93/3.73	4.78/4.20	4.20/3.94	4.61/4.12
Lower scleral show, right/left (mm)	1.66/0	0/0	0/0	0/0
Palpebral fissure height, right/left (mm)	9.72/7.46	5.74/5.60	6.30/5.96	6.02/5.92
Lateral canthal height, right/left (mm)	0.90/2.35	−1.55/2.59	1.19/1.64	0.98/2.56
Absolute bilateral lateral canthal height difference (mm)	1.45	4.14	0.45	1.58
Residual vertical gap on forceful closure (mm)	0	0	0	0

## Data Availability

The data supporting the findings of this study are available from the corresponding author upon reasonable request. Identifiable clinical images and videos are not publicly available because of patient privacy restrictions.

## Supplementary Materials

The following supporting information can be downloaded at https://www.mdpi.com/article/10.3390/jcm15176844/s1, Figure S1: Final-follow-up nasal endoscopy of the septal cartilage donor site. The healed septal mucosal incision, nasal septum, common nasal meatus, and inferior turbinate are labeled; the mucosa is healed without visible hypertrophic scarring or obvious donor-site deformity. Video S1: Postexcisional right lower eyelid–bicanthal defect; Video S2: Patient-specific reconstructive design combining a right transtemporal midface lift, a septal chondromucosal graft, and a bipedicled upper eyelid orbicularis myocutaneous flap.

## Abbreviations

The following abbreviations are used in this manuscript:

BCC	Basal cell carcinoma
HWTW	Horizontal white-to-white corneal diameter
MRD2	Marginal reflex distance 2
MRI	Magnetic resonance imaging
